# Osteogenic sarcoma of the breast arising in a cystosarcoma phyllodes: a case report and review of the literature

**DOI:** 10.1186/1752-1947-5-293

**Published:** 2011-07-07

**Authors:** Vinay Singhal, Chintamani, John M Cosgrove

**Affiliations:** 1 Department of Surgery, Bronx Lebanon Hospital Centre, Bronx, NY 10457, USA; 2 Department of Surgery, Vardhman Mahavir Medical College and Safdarjang Hospital, New Delhi, India

## Abstract

**Introduction:**

Primary tumors of the breast containing bone and cartilage are extremely rare, and an osteogenic sarcoma arising from a cystosarcoma phyllodes is exceptional.

**Case presentation:**

A 40-year-old Indian woman presented with a breast mass which was diagnosed as osteosarcoma of the breast on biopsy. Our patient was treated with a simple mastectomy after excluding the presence of skeletal primary and extra-mammary metastases. Final pathology showed a cystosarcoma phyllodes with signs of osteogenic sarcoma.

**Conclusion:**

Although osteogenic sarcomas of the breast are rare, they need to be distinguished from carcinosarcomas and metaplastic carcinomas as the management of the two differ.

## Introduction

Carcinoma is the most common malignancy of the breast. Sarcomas form a minority of breast neoplasms. Extra-skeletal osteosarcomas have been reported in many tissues of the body including thyroid gland, kidneys, bladder, colon, heart, testes and penis. In the breast it either occurs as a metaplastic differentiation of a pre-existing benign or malignant tumor; or *de novo *from normal breast tissue. We present a case of osteogenic sarcoma arising in a cystosarcoma phyllodes of the breast.

## Case presentation

**Figure 1:**
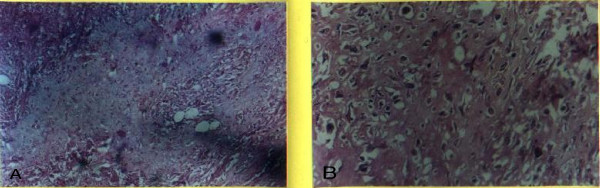
**Hyaline matrix with chondroid cells and osteoid cells**. (A) 100 × H&E stain hyaline matrix with chondroid cells. (B) 200 × H&E stain showing osteoids.

**Figure 2:**
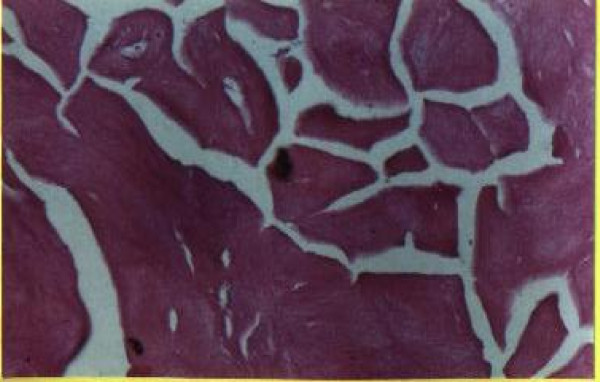
**100 × H&E stain showing cystosarcoma phyllodes with area of necrosis**.

A 40-year-old Indian woman presented to our outpatient department with complaints of a lump in her left breast noted four months prior to presentation. The lump gradually increased in size and was non-tender. There was no history of nipple discharge. The patient denied any hormonal therapy or family history of breast disease. A physical examination found our patient to be obese and in no acute distress. A breast examination showed her left breast to be pendulous with a 6 cm × 5 cm × 6 cm irregular, firm mass fixed to the overlying skin in the midline above her left nipple. There was no nipple discharge or skin dimpling. There were no palpable axillary lymph nodes. Her right breast and axilla were found to be normal. The remainder of the physical examination was noncontributory. A mammogram of her breast showed a well-defined mass measuring 5 cm with lobulated margins and areas of calcification closely resembling bone. In addition, fine egg shell calcification around the tumor was also noted. A core needle biopsy was taken from the breast lump which was reported as osteosarcoma of the breast. Computed tomography of the chest and abdomen, serum alkaline phosphatase and a bone scan were all within normal limits. Our patient was prepared for surgery and a simple mastectomy was performed. The histopathological examination revealed a tumor consisting of highly pleomorphic oval- to spindle-shaped cells arranged in sheets and bundles separated by fibrocollagenous tissue (Figure 1). The tumor cells had hyperchromatic nuclei and some showed mitotic figures. The stroma showed lymphocytic infiltration and areas of osteoid formation (Figure 2). Areas of hyaline matrix were intermingled, with vacuolated cells showing cartilaginous differentiation. The picture was suggestive of osteosarcoma of the breast in a pre-existing phyllodes tumor showing areas of chondroid differentiation. The immediate post-operative period was uneventful and our patient was discharged on the fifth post-operative day after removal of the suction drain. Five years post-operatively our patient is doing well and is in regular follow-up.

## Discussion

Sarcomas of the breast are relatively rare neoplasms accounting for less than 1% of breast malignancies [1]. Histological examination shows the majority to be fibrosarcomas, malignant fibrous histiocytomas and undifferentiated high grade sarcomas [2]. Tumors of the breast containing bone and cartilage can be divided into four groups: intra-ductal papilloma with stromal metaplasia; cystosarcoma phyllodes; stromal sarcoma; and adenocarcinoma with metaplasia [3]. The mechanism of formation of bone and cartilage differs in the above noted groups. In the lesions classified as adenocarcinoma with metaplasia, there is metaplasia of the epithelial cells to cartilage or bone while in the cystosarcoma and intra-ductal papilloma there is metaplasia of the stromal cells [4]. Pathological bone formation in the breast tissue may be the result of inter-membranous ossification and the marrow is not observed [5,6]. Extra-osseous osteosarcomas have also been reported in thyroid gland, kidney, urinary bladder and uterus [7]. Overall, mammary osteosarcomas are biologically aggressive tumors characterized by early recurrences and hematogenous metastasis, frequently to the lungs [8]. Optimal management should include total excision of the neoplasm with an adequate margin for control of local disease. A simple mastectomy may be indicated to ensure complete excision of large tumors with cryptically infiltrative margins [2]. Axillary lymph node dissection is not indicated in the setting of clinically negative nodes. Although the role of adjuvant therapy is unclear, several studies involving a small number of patients suggest that adjuvant chemotherapy may be of value in patient management [8]. Distinguishing metaplastic carcinoma and carcinosarcoma from osteosarcoma of the breast is important, because the former necessitates treatment as primary breast cancer. Finally, although the breast is an unusual site of metastases, it is necessary to exclude the possibility of a metastatic lesion, as well as primary osseous osteosarcoma, before establishing the diagnosis as osteosarcoma of the breast.

## Conclusion

Although osteogenic sarcoma of the breast is rare, it needs to be distinguished from carcinosarcoma and metaplastic carcinoma as the management differs.

## Competing interests

The authors declare that they have no competing interests.

## Consent

Written informed consent was obtained from the patient for publication of this case report and any accompanying images. A copy of the written consent is available for review by the Editor-in-Chief of this journal.

## Authors' contributions

VS was the surgical assistant, compiled the data and prepared the manuscript. C was the surgeon who operated on the patient. JMC helped in preparation of the final manuscript. All authors read and approved the final manuscript.
